# Epidemiology, species distribution, antifungal susceptibility and mortality risk factors of candidemia among critically ill patients: a retrospective study from 2011 to 2017 in a teaching hospital in China

**DOI:** 10.1186/s13756-019-0534-2

**Published:** 2019-05-29

**Authors:** Zengli Xiao, Qi Wang, Fengxue Zhu, Youzhong An

**Affiliations:** Peking University People’s Hospital, No. 11 Xizhimen South Street, Xicheng District, Beijing, 100044 People’s Republic of China

**Keywords:** Candidemia, Species distribution, Antifungal susceptibility, Risk factors

## Abstract

**Background:**

Candidemia is still a common life-threatening disease and causes significant morbidity and mortality, especially in critically ill patients. We conducted this study to analyze the epidemiology, clinical characteristics, species distribution, antifungal susceptibility and mortality risk factors of candidemia in an intensive care unit.

**Methods:**

We retrospectively analyzed patients with candidemia in the intensive care unit of our hospital from 2011 to 2017. The clinical characteristics, including clinical and laboratory data, antibiotic therapies, underlying conditions, and invasive procedures and outcomes, were analyzed. We also performed a logistic regression analysis to identify the independent risk factors for mortality.

**Results:**

In this six-year retrospective study, we identified 82 patients with candidemia. The median age of the patients was 76 years (range, 26 years to 91 years), and 50 of the patients (61%) were male. *Candida albicans* was the most common fungal species (38/82, 46.3%), followed by *Candida parapsilosis* (16/82, 19.5%), *Candida glabrata* (13/82, 15.9%), and *Candida tropicalis* (12/82, 14.6%). Most isolates were susceptible to the antifungal agents. The all-cause mortality rate was 51.2%. In binary logistic regression analysis, the worst Glasgow coma score (GCS), PaO_2_/FiO_2_ ratio (P/F ratio), and mean arterial pressure (MAP) within three days after admission were independent risk factors for mortality.

**Conclusions:**

*Candida albicans* was the most frequently isolated fungal species. Most isolates were susceptible to the antifungal agents. The worst GCS score, P/F ratio, and MAP within three days after admission were independent risk factors for mortality due to candidemia in critically ill patients.

## Background

Invasive candidiasis (IC) has become a substantial threat to public health. IC affects more than 250,000 people every year and is associated with a mortality rate exceeding 70% [1–3].

Traditionally, IC has been associated with immunocompromised states, chronic inflammatory diseases and chronic immunosuppressive conditions. The use of broad-spectrum antibiotics, any pre-existing cause of immunosuppression, recent surgery and indwelling central venous catheters (CVC), particularly those for total parenteral nutrition, are all associated with IC [4, 5]. Therefore, the number of patients at risk of fungal infection is rising in intensive care units (ICUs), and as the third most common cause of infection in the ICU worldwide, accounting for 17% of infections, it has become a growing concern for doctors [6–8].

Thus far, more than 17 different *Candida* species have been identified as causative pathogens of bloodstream infections (BSIs), and *Candida albicans* is the dominant and most extensively studied pathogen [9, 10]. However, the proportion of non-*Candida albicans* strains has increased rapidly in recent years [11]. In general, more than 90% of IC are caused by *C. albicans*, *C. tropicalis*, *C. parapsilosis*, *C. glabrate* and *C. krusei* [12, 13].

In the ICU, the treatment of IC remains a challenge. Systemic antifungal therapy is used in up to 7.5% of ICU patients, although two-thirds of these patients have no documented IC [14]. The current guidelines also recommend empiric antifungal treatment, although it often fails to confer any benefit on ICU patients [15, 16]. Therefore, the availability of local epidemiological data could help improve antifungal stewardship.

The aim of this study was to describe the epidemiology, clinical characteristics, species distribution, antifungal susceptibility and mortality risk factors for of candidemia in an ICU in China. We believe that knowledge of IC epidemiology, including geographical differences, is an important guide to prescribing practices and health policies and thus has far-reaching clinical implications [17].

## Methods

### Patients and setting

From March 2011 to September 2017, all patients with candidemia reported by the microbiological department of the ICU of Peking University People’s Hospital in China were retrospectively identified and analyzed. There are 41 beds in this general ICU. The medical records of all patients due to *Candida* infection were reviewed, and the following information was collected: age, sex, patient source (medical/surgical ward), underlying conditions (diabetes, hypertension, chronic obstructive pulmonary disease, chronic cardiac disease, chronic liver disease, solid tumor, hematological malignancy, chronic renal dysfunction, surgery within 1 month), the worst GCS score within 3 days after admission to the ICU, previous treatment (antifungal treatment, steroid therapy, antibiotic therapy), the worst laboratory data within 3 days after admission to the ICU (P/F ratio, hemoglobin level, neutrophil count, white blood cell count, temperature, serum total protein level, serum albumin level, MAP), previous invasive procedures (central venous catheter, urinary tract catheter, total parenteral nutrition), and the worst Sequential Organ Failure Assessment (SOFA) score within 3 days after admission to the ICU (length of ICU, length of hospital stay, and duration of mechanical ventilation).

### Definitions

We defined candidemia as the isolation of *Candida* species from at least one blood culture in patients with symptoms or signs of a systemic infection. Anemia was defined as a hemoglobin level < 70 g/l. Neutropenia was defined as an absolute neutrophil count < 1.5*10^9^/l. Hypoproteinemia was defined as a total protein level < 60 g/dl or serum albumin level < 25 g/dl. Empirical antifungal therapy was defined as the administration of antifungals in patients with refractory pyrexia and other risk factors for IC before the results of antifungal sensitivity testing were obtained.

### Microbiology and antifungal susceptibility testing

Isolates were collected from blood cultures using the ALERT 3D automated system (bioMérieux, Marcy l’Etoile, France) and were identified by the Vitek 2 automated system (bioMérieux, Marcy l’Etoile, France). Antifungal susceptibility testing was performed using the ATB FUNGUS 3 strip (bioMérieux, Marcy l’Etoile, France) in accordance with the manufacturer’s instructions. Five antifungal drugs with different concentrations, namely, 5-flucytosine, amphotericin B, fluconazole, itraconazole and voriconazole, were tested. After incubation at 35 °C for 24 h, the strips were read visually to determine the scores. The MICs were interpreted according to species-specific Clinical & Laboratory Standards (CLSI) M27-A3 breakpoints. *C. krusei* ATCC 6258 and *C. parapsilosis* ATCC 2019 were used as quality controls [18].

### Statistical analysis

Data were analyzed with SPSS software version 21.0. The count data were described by case number (n), and the difference between groups was tested by the Chi-square test or Fisher’s exact test. The Shapiro-Wilk normality test showed that the measurement data did not conform to a normal distribution (*p* < 0.05). Therefore, the measurement data in this study were described by P50 (P25,P75), and a nonparametric rank sum test was used for intergroup comparison. Factors with a *p* < 0.05 in univariate tests were analyzed with a binary logistic regression model to identify the independent risk factors. The difference was statistically significant when *p* < 0.05.

## Results

### Patient characteristics and distribution of *Candida* species

Eighty-two patients with candidemia were identified over a 6-year period. The median age of the patients was 76 years (range, 26 years to 91 years), and 50 patients (61%) were male. Approximately 65.9% of the patients came from the surgical ward. Surgery within 1 month (58.5%), hypertension (43.9%), and solid tumors (36.6%) were the most common underlying conditions, followed by diabetes (32.9%), chronic cardiac disease (22%), chronic renal dysfunction (20.7%), chronic liver disease (11%), hematological malignancy (6.1%), and chronic obstructive pulmonary disease (COPD) (2.4%). The median GCS score of these patients was 11. In total, 27 (32.9%) patients had received previous antifungal treatment. Fluconazole was the most frequently used empirical antifungal treatment (40/64, 62.5%). The patients’ laboratory data and previous invasive procedures are also shown in Table 1.

**Table 1:** Baseline characteristics of 82 patients diagnosed with IC in the ICU

	N (%) or P_50_ (P_25_,P_75_)	Mortality status (all-cause)			
		Survived (*n* = 40)	Did not survive (*n* = 42)	Z/χ^2^/ Fisher’s exact test	*P*
Age (years)	76.0 (65.8, 82.0)	76.0 (61.3, 81.8)	78.0 (67.0, 84.0)	−0.673^#^	0.501
Males	50 (61.0)	26 (65.0)	24 (57.1)	0.532^*^	0.466
Hospital admission					
Medical ward	28 (34.1)	14 (35.0)	14 (33.3)	0.025^*^	0.874
Surgical ward	54 (65.9)	26 (65.0)	28 (66.7)		
Underlying conditions					
Diabetes	27 (32.9)	12 (30.0)	15 (35.7)	0.303^*^	0.582
Hypertension	36 (43.9)	14 (35.0)	22 (52.4)	2.513^*^	0.113
Chronic cardiac disease	18 (22.0)	7 (17.5)	11 (26.2)	0.903^*^	0.342
Chronic liver disease	9 (11.0)	5 (12.5)	4 (9.5)	0.006^*^	0.938
Solid tumor	30 (36.6)	15 (37.5)	15 (35.7)	0.028^*^	0.867
COPD	2 (2.4)	0 (0.0)	2 (4.8)	-^Δ^	0.494
Hematological malignancy	5 (6.1)	2 (5.0)	3 (7.1)	0.000^*^	1.000
Chronic renal dysfunction	17 (20.7)	4 (10.0)	13 (31.0)	5.473^*^	**0.019**
Surgery	48 (58.5)	25 (62.5)	23 (54.8)	0.505^*^	0.477
GCS score	11.0 (9.0, 15.0)	11.0 (11.0, 15.0)	10.0 (6.0, 11.0)	−4.225^#^	**0.000**
Previous treatment					
Antifungal treatment	27 (32.9)	12 (30.0)	15 (35.7)	0.303^*^	0.582
Caspofungin	22 (26.8)	11 (27.5)	11 (26.2)	0.018^*^	0.894
Fluconazole	39 (47.6)	19 (47.5)	20 (47.6)	0.000^*^	0.991
Amphotericin B	2 (2.4)	0 (0.0)	2 (4.8)	-^Δ^	0.494
Voriconazole	32 (39.0)	18 (45.0)	14 (33.3)	1.172^*^	0.279
Previous steroid therapy	25 (30.5)	10 (25.0)	15 (35.7)	1.110^*^	0.292
Previous antibiotic therapy	63 (76.8)	31 (77.5)	32 (76.2)	0.020^*^	0.888
Laboratory data					
P/F ratio	256.1 (188.4, 337.3)	290.5 (241.5, 370.8)	224.0 (130.0, 270.0)	−3.553^#^	**0.000**
Anemia (HGB < 70 g/l)	27 (32.9)	10 (25.0)	17 (40.5)	2.222^*^	0.136
Neutropenia (< 1 months)	7 (8.5)	2 (5.0)	5 (11.9)	0.523^*^	0.470
WBC > 20*10^9^/l	26 (31.7)	11 (27.5)	15 (35.7)	0.638^*^	0.424
Temperature > 38 °C	68 (82.9)	31 (77.5)	37 (88.1)	1.624^*^	0.202
Hypoproteinemia	81 (98.8)	39 (97.5)	42 (100.0)	-^Δ^	0.488
MAP, mean (SD)	76.7 (66.5, 90.0)	85.5 (73.3, 94.5)	68.0 (57.0, 78.3)	−4.134^#^	**0.000**
Previous invasive procedures					
Central venous catheter	13.0 (5.5, 27.0)	13.0 (6.0, 25.5)	14.0 (4.5, 29.5)	−0.454^#^	0.650
Urinary tract catheter	13.0 (5.0, 27.0)	12.5 (5.0, 24.0)	17.0 (5.5, 35.5)	−1.026^#^	0.305
Total parenteral nutrition	10.5 (3.8, 21.3)	8.5 (3.3, 15.8)	13.0 (4.5, 27)	−1.023^#^	0.307
Mechanical ventilation	3.5 (1.0, 10.3)	2.5 (1.0, 9.5)	5.50 (1.0, 15.0)	−1.514^#^	0.130
SOFA score	8.0 (4.0, 11.0)	5.0 (4.0, 8.0)	10.0 (8.5, 14.0)	−4.628^#^	**0.000**
Outcome (days)					
ICU length of stay	16.0 (4.8, 51.8)	16.0 (5.3, 36.3)	14.5 (3.0, 66.0)	−0.223^#^	0.823
Hospital length of stay	45.0 (27.8, 81.3)	40.0 (28.0, 98.0)	49.0 (26.5, 81.0)	−0.014^#^	0.989
Mechanical ventilation	8.5 (1.8, 23.3)	5.0 (1.0, 11.0)	11.5 (3.0, 37.5)	−2.461^#^	**0.014**

^*^: Chi-square test; ^#^: rank sum test; ^Δ^: Fisher’s exact test

P_50_ (P_25_,P_75_): the quartile summary is viewed as P25, P50, and P75. For P50, there is a 50% chance that the mean power production will not be reached at any given time

### Distribution of *Candida* spp. causing BSIs

**Fig. 1:**
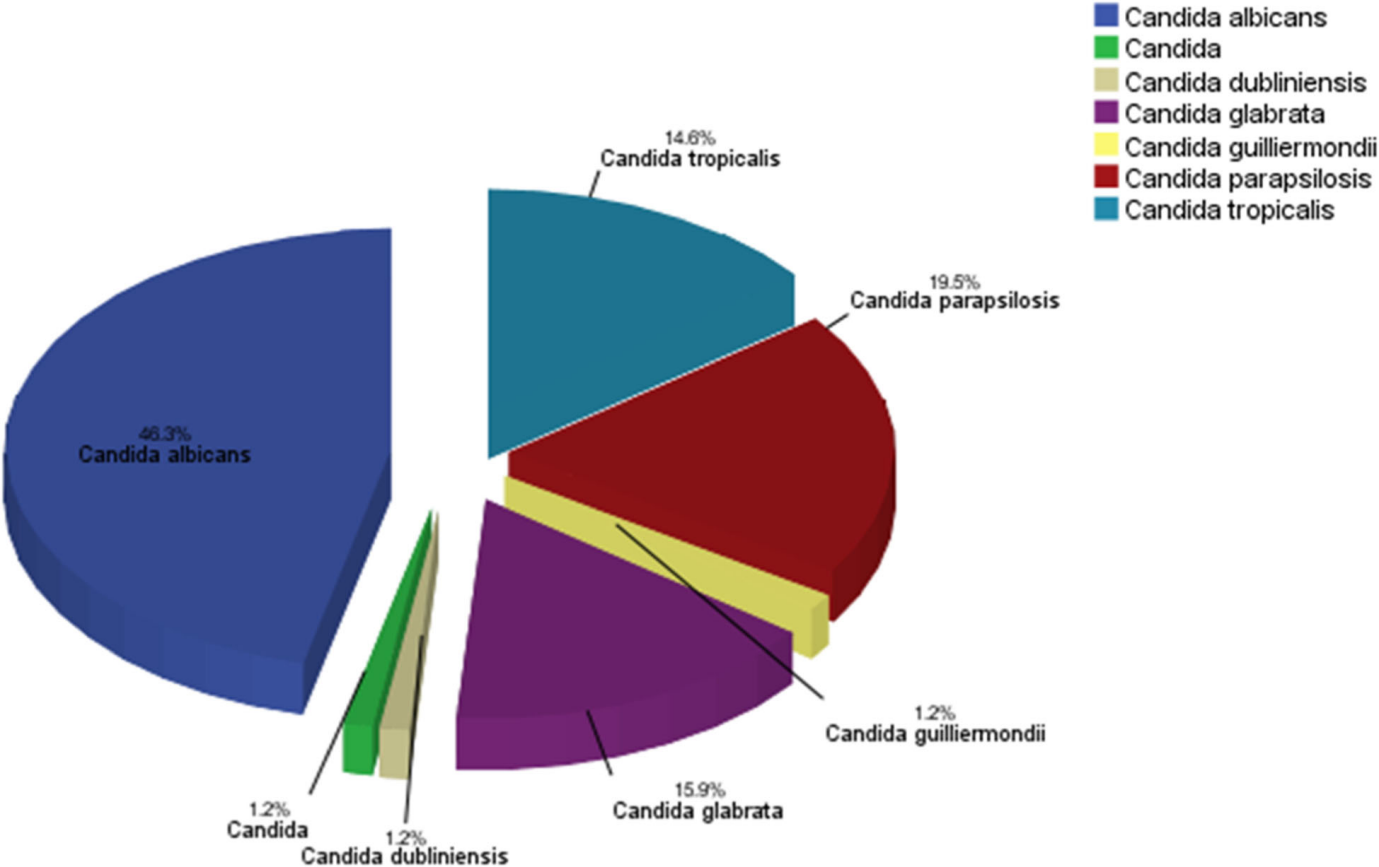
Distribution of Candida species responsible for candidiasis (*n* = 82). *Candida albicans* (*n* = 38), *Candida parapsilosis* (*n* = 16), *Candida glabrata* (*n* = 13), *Candida tropicalis* (*n* = 12), *Candida dubliniensis* (*n* = 1), *Candida guilliermondii* (*n* = 1), *Candida* spp. (*n* = 1)

*Candida albicans* was by far the most prevalent fungal species (46.3%), followed by *Candida parapsilosis* (19.5%), *Candida glabrata* (15.9%), *Candida tropicalis* (14.6%), *Candida dubliniensis* (1.2%), *Candida guilliermondii* (1.2%), and *Candia* spp. (1.2%) (Fig. 1).

### Susceptibilities of the isolates

**Fig. 2:**
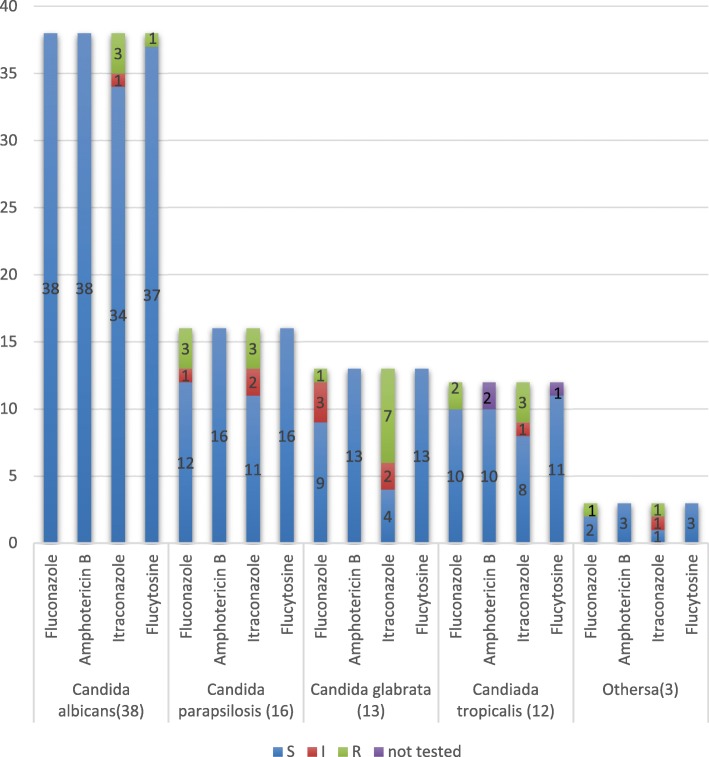
In vitro susceptibility data for the Candida spp. ^a^ Others include *Candida dubliniensis* (*n* = 1), *Candida guilliermondii* (*n* = 1), and Candia (*n* = 1). S = susceptible; I = intermediate; R = resistant

The in vitro antifungal susceptibility of the isolated *Candida* species is presented in Fig. 2. Overall, most isolates were susceptible to the antifungals. *C. albicans, C. tropicalis,* and *C. parasilosis* were highly susceptible to all antifungal agents, whereas the other *Candida* species had low levels of susceptibility to fluconazole and itraconazole. Itraconazole had the highest drug resistance rate, while no species showed resistance to amphotericin B.

### Outcomes and risk factors for mortality

The all-cause in-hospital mortality rate of the 82 patients was 42/82 (51.2%). In univariate analyses, increased mortality was associated with chronic renal dysfunction, GCS score, P/F ratio, MAP, and SOFA score, as shown in Table 1. The median ICU length of stay and hospital length of stay were 16 days and 45 days respectively. The median duration of mechanical ventilation was 8.5 days and the survived group was significant less than died group (Table 1). In binary logistic regression analysis, the worst value of GCS score, P/F ratio, MAP within three days after admission were independent risk factors for mortality (Table 2).

**Table 2:** Risk factors for mortality

	B	Wald	OR	CI	P
Chronic renal dysfunction	1.458	2.664	4.299	0.746–24.772	0.103
GCS score	−0.256	4.408	0.774	0.610–0.983	0.036
P/F ratio	−0.006	4.862	0.994	0.988–0.999	0.027
MAP	−0.046	5.220	0.955	0.919–0.994	0.022
SOFA score	0.114	1.431	1.121	0.930–1.352	0.232

B:coefficient estimates; Wald: Chi-square value; OR: Odds ratio; CI;Confidence interval

## Discussion

We report a 6-year retrospective study of candidemia at the Peking University People’s Hospital, a teaching hospital in China. We not only focused on the main epidemiological characteristics, such as risk factors and antifungal agent use, but we also obtained a complete overview of candidemia, including *Candida* species identification, antifungal resistance determination and patient outcome analysis.

The mean age of patients in our study was older than the mean ages in previous studies, possibly because we mainly focused on critically ill patients; critically ill patients are often older than other patients [2, 19, 20]. Similar to other studies, *C. albicans* was the most common species causing candidemia, followed by *C. parapsilosis, C. glabrata,* and *C. tropicalis* [21–25]. This may be due to increasing numbers of surgeries, the aging of the population, and the increasing use of antifungal agents.

Susceptibility testing for antifungal drugs was performed for all isolates of *Candida* species. In our study, fluconazole was active against *C. albicans,* but a trend towards increased resistance or the emergence of naturally resistant species was observed among other *Candida* spp., implying that fluconazole could be used in patients with *Candida albicans* as a first-line agent [26]. For patients infected with other *Candida* spp., fluconazole should be used according to the results of the susceptibility test. Amphotericin B demonstrated excellent activity against all *Candida* species. Resistance to itraconazole was relatively more common in all *Candida* species, which was consistent with the findings of other studies [27]. These findings should be taken into consideration when establishing antifungal treatment strategies.

Invasive *Candida* infection in the ICU is an increasing concern due to its high associated mortality rate and resource consumption. According to previous studies, invasive *Candida* infection is associated with mortality rates of 35–80% in the ICU [28–31]. The all-cause mortality of patients with candidemia in our study was 51.2%, which is within the range of previously reported mortality rates [32, 33]. The mean time between diagnosis and death was 16.7 days. The ICU length of stay and hospital length of stay for candidemia patients were much longer than those for general ICU patients [34].

In univariate analyses, increased mortality was associated with chronic renal dysfunction, GCS score, P/F ratio, MAP, and SOFA score, as shown in Table 1. The median ICU length of stay and hospital length of stay were 16 days and 45 days, respectively. The median duration of mechanical ventilation was 8.5 days, and the duration was significantly shorter in the surviving group was significantly less than the nonsurviving group (Table 1). In binary logistic regression analysis, the worst GCS score, P/F ratio, and MAP within 3 days after admission were independent risk factors for mortality.

In our study, most of the patients had long durations of central venous catheterization, urinary tract catheterization, and mechanical ventilation, which have been shown to be responsible for fungal infections [10, 35, 36]. In our study, the statistical analysis failed to confirm an association between these invasive procedures (CVC, urinary catheter) and mortality. The reason may be that 91.5% of the patients in our study continuously had a central venous catheter (95% for urinary catheter) in the ICU, making the groups of patients without a central catheter or without a urinary catheter too small for comparison. A previous study showed that catheter removal could reduce the incidence of candidemia. Therefore, if these catheters are suspected to be the source of candidemia, we should try to decrease the utilization of these invasive devices and remove these catheters as early as possible [14, 37]. In our study, 32.9% of patients received empirical antifungal therapy, which was similar to the result of a previous study. Current guidelines recommend empiric antifungal therapy; however, this often fails to provide any benefit to ICU patients and may result in significant overtreatment [16].

The limitations of this study must be acknowledged. This was a single-center retrospective study, so the results may not be generalizable to all patients with candidemia. The epidemiological findings in our institution will pave the way for more in-depth studies and help us establish better antifungal stewardship in our hospital.

## Conclusion

*C. albicans* was the most frequently isolated fungal species. Most isolates were susceptible to the antifungal agents. The worst GCS score, P/F ratio, and MAP within three days after admission were independent risk factors for mortality due to candidemia among critically ill patients. Further multicenter studies in different geographical regions on candidemia in critically ill patients should be conducted to help intensive care specialists assess the distribution and trends in their patients with suspected fungal infections.

## Abbreviations

BSI: Bloodstream infection
CLSI: Clinical and laboratory standards
COPD: Chronic obstructive pulmonary disease
CVC: Central venous catheter
GCS: Glasgow Coma Scale
IC: Invasive candidiasis
ICU: Intensive care unit
MAP: Mean arterial pressure
P/F ratio: PaO_2_/FiO_2_ ratio
SOFA: Sequential Organ Failure Assessment
